# Quantitative Risk Evaluation of Adventitious Agents in Heparin

**DOI:** 10.1055/s-0043-1768946

**Published:** 2023-05-21

**Authors:** John Raedts, Edwin Kellenbach

**Affiliations:** 1 Aspen Oss B.V., Oss, The Netherlands

**Keywords:** heparin, adventitious agents, viral safety, prion safety, virus reduction, prion reduction

## Abstract

Heparin is typically extracted from domestic pigs, which may carry zoonotic adventitious agents. Prion and viral safety cannot be assured by testing the active pharmaceutical ingredient itself; instead for the evaluation of the adventitious agent (i.e., viruses/prions) safety of heparin and heparinoid (e.g., Orgaran or Sulodexide) therapeutics, a risk assessment is required. An approach is presented which provides a quantitative estimation of the worst-case potential residual adventitious agent (i.e., GC/mL or ID _50_ ) present in a maximum daily dose of heparin. This estimation is based on the input (determined by prevalence, titer, and amount of starting material to prepare a maximum daily dose) and validated reduction by the manufacturing process, resulting in an estimation of the worst-case potential level of adventitious agent present in a maximum daily dose. The merits of this quantitative, worst-case approach are evaluated. The approach described in this review provides a tool for a quantitative risk evaluation of the viral and prion safety of heparin.

## Introduction

The glycosaminoglycan heparin sodium is a parenteral anticoagulant, which typically is isolated from the intestines of domestic pigs. 1 Heparin sodium is the number two biological drug globally and has been used as an anticoagulant for over 100 years. 2 The two main challenges associated with extracted drugs such as heparin are supply (approximately 1,000,000,000 pigs are required annually to meet the global heparin need) and contamination with *i.a.* adventitious agents such as viruses and prions which may result in zoonotic disease. 3 4 5 In the wake of the coronavirus disease 2019 (COVID-19) pandemic, awareness of zoonotic viruses has risen. An animal-derived product that is administered by injection should be scrutinized for zoonotic agents to avoid iatrogenic infections. Prion and viral safety cannot be assured by testing the active pharmaceutical ingredient (API) itself, as is the case for bacteria, yeast, and molds as well as endotoxins because the analytical methods lack the sensitivity to assure prion and viral safety. A safety/quality by design strategy is therefore chosen, combining an estimation of the worst-case viral/transmissible spongiform encephalopathy (TSE) load of a worst-case daily dose with the viral and prion reduction of process steps determined at the laboratory scale. Here, we discuss the strategies developed to assess the prion and viral safety. Several model calculations are given for clarity's sake.

## Adventitious Agents

The World Health Organization defines adventitious agents as microorganisms, viruses, and TSE agents unintentionally introduced into the manufacturing process of a biological medicinal product. 6 Adventitious agents may be introduced in the manufacturing process via different routes, although typically these are linked to the sourcing of the crude materials, in the case of heparin the intestines of domestic pigs. The presence of adventitious agents potentially may pose significant risks to the quality and safety of biological drugs.

## Risk-Based Approach to Assess Adventitious Agent Risk

The evaluation of prion and viral safety risk assessments has several strategies in common:

- The worst-case prion and viral load is determined by multiplying the prevalence with the titer and the amount of starting material (porcine intestines) required to prepare a maximum dose of unfractionated heparin (UFH).
- Prevalence and titer for domestic pigs in the regions where the starting material is sourced are used. 7
- A worst-case approach in the calculation of the prion and viral load and the prion and viral reduction: highest number for prevalence and titer and conversion; lowest values for reduction by the manufacturing process.
- Reduction by the manufacturing process is determined in a validated downscale by spiking at a known level at the *start* of the process followed by the determination of the level (during and) *after* the performance of the process. The latter is usually performed by specialized CROs because of the safety restrictions associated with pathogenic viruses and prions.
- Except for the reduction validated steps, all other manufacturing process steps are assumed *not* to reduce the prion and viral load, as a worst-case approach.

## Prevalence of Viruses in Domestic Pigs in the Sourcing Regions

The first element to consider is to restrict the risk assessment to zoonotic viruses in domestic pigs in the sourcing region(s). The web sites of the European Food Safety Authority, 8 the U.S. Centers for Disease Control and Prevention, 9 and the World Organization for Animal Health 10 offer authoritative, up-to-date information about the geographic distribution of zoonotic diseases, but alternative web sites are available. 11 12 A recent article gave a useful summary of the zoonotic viruses in domestic pigs globally. 7 Typical examples of zoonotic viruses to consider in a porcine-derived heparin virus risk assessment are hepatitis E virus and influenza virus. More detailed information on specific viruses can be found in the scientific literature. If a virus does not occur in the sourcing region(s) and/or is not zoonotic, there is no reason to include the virus in a quantitative risk assessment because it does not pose a risk for infection and only adds in a negligible way to the residual protein and nucleotide impurities. For instance, African swine fever is not infectious to man 13 and COVID-19, conversely, is not infectious to domestic pigs. 14 15 16 17 18 19 20 Some viruses, such as the Ebola virus, 21 are limited to local, incidental breakouts, and are not endemic in domestic pigs. These viruses should be dealt with case by case and do not lend themselves to a quantitative risk assessment as discussed in this paper.

## Virus Titer in the Sourced Material

After the prevalence of zoonotic viruses in the sourcing regions has been established, the titer/number of infectious virus particles in the sourced material needs to be determined as the next step in the calculation of the viral load of the process. These data are either obtained from the literature or determined experimentally.

For most viruses commonly found in domestic pigs, literature data are available reporting typical virus titers in infected animals. For each virus, the viral load in porcine intestinal material is estimated from data as found in scientific publications and assumptions made thereof, using a worst-case approach. In case viral load data are reported for porcine intestinal mucosa, results are used as reported. In case these data are not available/not found, but viral load data are available for other porcine specimen types, the result obtained with the specimen type with the highest reported viral load result is applied to the potential viral load in porcine intestinal mucosa unless stated differently (e.g., in case it is obvious and motivated that lower values apply for mucosa specimens). In case these data are also not available, but viral load data are reported for other species (preferably human) and/or other specimen types than intestinal mucosa, the reported results will be applied for porcine intestinal mucosa unless stated differently. In case, no viral load data are available at all, but real-time polymerase chain reaction (PCR) values are reported, the amount of virus will be calculated from the PCR result based on the threshold cycle value.

An alternative approach may be based on experimental viral load data of the starting material as used in the heparin process. This requires a quantitative PCR method validated for the detection of one specific virus in heparin crude starting material. In case of virus negative starting material, the worst-case potential maximum virus input per batch of heparin can be estimated by multiplying the sensitivity limit (Limit of Detection expressed as IU/mL starting material) with the maximum amount of starting material per batch of heparin.

The corresponding estimated potential maximum virus input per batch of heparin is considered a conservative approach as RT-PCR uses viral genomes expressed in International Units (IU) as an indicator of the potential infectious virus on a 1-to-1 basis. This approach provides a worst-case scenario because the presence of a viral genome does not automatically implicate the presence of an infectious viral particle. In general, viruses can survive outside the cell for only a limited time. Outside the host, the infectivity of most viruses is inherently unstable.

## Prion and Viral Clearance Studies

The heparin manufacturing process consists of multiple process steps having the capacity to remove or inactivate viruses and TSE agents. The clearance of prions and viruses in the manufacturing process is generally based on the reduction of suitable models representing the contaminants of the host material. A typical model used for clearance studies for prion proteins is 263K hamster-adapted scrapie agent. For virus clearance studies, typically four model viruses are selected, representing all viruses: DNA/RNA-based genome, enveloped and non-enveloped viruses. The selection can further be augmented to include viruses of a variety of sizes and shapes of virus particles. In viral clearance studies spikes of these models into process intermediates and, subsequently, their removal or inactivation in the downstream processing step is validated.

In heparin processes, the multiple common unit operations can be considered for virus and prion removal/inactivation. An overview of the process step that may be considered is provided in Table 1 .

**Table 1:** Common unit operations in the heparin purification process to be considered for virus and prion removal/inactivation

Process step	Mode of action	Typical LRF	Ref.
Chromatography	Virus removal from the product stream	2–6 log _10_	35 36 37
Heat treatment	Virus inactivation by induction of structural changes in viral proteins	2–7 log _10_	38 39 40 41 42 43
Low and high pH	Virus inactivation by induction of structural changes in viral proteins	4–6 log _10_	35 36 37 44 45
Organic solvents	Virus inactivation by disruption of the virus envelop	4–6 log _10_	36 37 44 45 46 47 48 49
Precipitation	Virus removal from the product stream	2–5 log _10_	37
Oxidizing agent	Virus inactivation by induction of structural changes in viral proteins	3–5 log _10_	48 50
Virus filtration	Virus retention using nanofiltration	4–6 log _10_	35 36 37 44 45

Abbreviation: LRF, logarithmic reduction factor.

In addition, virus filtration can be introduced as a dedicated virus removal step. Heparin is an extremely stable molecule because of its inherent chemical stability 22 and because, not being a protein, its action does not depend on a higher-order structure. Heparin also is the most negatively charged biopolymer known. These qualities allow the isolation of relatively low amounts of heparin of about one daily dose of a few hundred milligrams per pig. 1 23 The harsh isolation conditions include exhaustive digestion by proteolytic enzymes, oxidation by strong chemical oxidizer such as potassium permanganate or hydrogen peroxide at elevated temperatures and pH, as well as exposure to high alcohol levels. 1 23 These conditions would result in the denaturation of therapeutic proteins, where the action depends on a higher-order structure stabilized, in part, by weak, noncovalent forces. In heparin, however, the therapeutic action relies on the primary structure dictated by strong covalent bonds. Further processing by conversion of unfragmented heparin to low molecular weight heparin (LMWH) may also involve virus- and prion-reducing steps. These harsh conditions result in efficient inactivation/removal of adventitious agents yielding safe medicinal products. Briefly, both virus and prion clearance validation is performed at a laboratory scale at a specialized CRO by spiking the material with a known amount of virus/prion, performing the validated, downscaled process step, and determining the residual virus/prion. The ratio between the spiked amount, and the residual amount of virus/prion after process step execution, is the reduction factor, which is usually expressed as a Log _10_ providing a logarithmic reduction value or logarithmic reduction factor.

## Conversion

Finally, the amount of sourced material required to manufacture a worst-case daily dose heparin sodium needs to be obtained. For heparin, 60,000 units is the maximum daily dose found in the literature. 24 Using a worst-case activity of 180 IU/mg on dried substance, this corresponds to a mass of 333 mg, daily. From the validation of the manufacturing process, from sourced material to API, again worst case, the lowest overall unit yield is used to calculate the amount of sourced material required to produce a single worst-case dose of 333 mg heparin sodium.

It should also be stressed that UFH is frequently depolymerized to yield LMWHs. The depolymerization processes often comprise process steps having potential which can be exploited to validate viruses and/or prion reduction. 25

## Calculation

Using all the above-mentioned input, the theoretical worst-case potential residual risk can be calculated as follows:

X = M × P × T/R

in which the amount of sourced material required to manufacture a worst-case daily dose heparin sodium (M) is *multiplied with* the prevalence of the virus in domestic pigs in the sourcing area (P) *multiplied with* the titer of the virus (T) *divided by* the validated reduction factor in the manufacturing process (R) *provides* the theoretical worst-case potential virus level present in a worst-case daily dose of heparin (X).

**Fig. 1:**
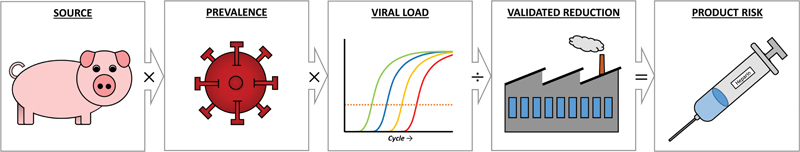
Graphical abstract quantitative risk evaluation of viruses in heparin.

This calculation is graphically summarized in Fig. 1 .

As an example, the following fictitious quantitative risk evaluation is included concerning the risk of influenza virus in heparin sodium manufactured from mucosa. For the sake of this calculation, the amount of material (M) required for one dose of heparin sodium is assumed to roughly correspond to the small intestine of one pig which we set at 1 kg. 26 The prevalence (P) of influenza virus in European pigs is 31% 27 with a titer (T) of 3 × 10 ^8^ viral particles per kg material. 28 Assuming four process steps orthogonally contributing to virus reduction, each contributing in a 3 log _10_ reduction, the total reduction factor (R) is (3 log _10_ ) 4 = 12 log _10_ . Combining these individual parameters, the risk per dose of heparin equals 1 kg × 31% ×  3 × 10 ^8^ viral particles per kg/12 log _10_ reduction = 0.000093 viral particles per maximum daily dose of heparin.

## Discussion

The calculations yield a deceptively simple number representing the theoretical worst-case potential virus/TSE level present in a worst-case daily dose of heparin. But there is a *caveat* : because of the many, often unrealistic but inevitable worst-case assumptions, the number is likely to be a (gross) overestimation of the actual virus/TSE levels present. For example, the virus and prion reduction studies often fail to detect any adventitious agent after the execution of the process step. However, because of the limited sensitivity of the detection (the limit of detection/limit of quantitation are > 0 per definition), this often results necessarily in an underestimation of the reduction yielding an overestimation of the virus and prion levels. Therefore, the calculated worst-case risks are more suited to compare the relative safety of processes rather than to express a realistic estimate of the absolute risk.

Furthermore, specifically for TSE calculations should be considered that:

- Classical BSE prevalence in cattle worldwide has dropped precipitously since the end of the 20th century 29 and nowadays the prevalence is extremely low. 30 Accordingly, the reintroduction of bovine heparin is considered. 31 32
- There is no scientific proof that scrapie can be transmitted from animals to humans under real-life conditions. 33
- Heparin sodium is isolated from porcine intestines. Pigs are not TSE-relevant animal species as defined by the EMA's Note for Guidance. 34
- TSE infectivity is usually evaluated by intracranial injection, which is not the customary administration route for heparin. This gives again the worst-case, overestimated value of infectivity.

## Conclusion

The discussed quantitative adventitious agent risk assessment approach integrates the various factors contributing to the theoretical worst-case potential virus/TSE level present in a worst-case daily dose of heparin. However, the calculated outcome should be interpreted with care because the approach inherently results in a (gross) overestimation of the potential virus/TSE level present in a worst-case daily dose of heparin.
